# *Sphingomonas* sp. Cra20 Increases Plant Growth Rate and Alters Rhizosphere Microbial Community Structure of *Arabidopsis thaliana* Under Drought Stress

**DOI:** 10.3389/fmicb.2019.01221

**Published:** 2019-06-05

**Authors:** Yang Luo, Fang Wang, Yaolong Huang, Meng Zhou, Jiangli Gao, Taozhe Yan, Hongmei Sheng, Lizhe An

**Affiliations:** ^1^Ministry of Education Key Laboratory of Cell Activities and Stress Adaptations, School of Life Sciences, Lanzhou University, Lanzhou, China; ^2^The College of Forestry, Beijing Forestry University, Beijing, China

**Keywords:** *Arabidopsis thaliana*, plant growth-promoting rhizobacteria, *Sphingomonas* sp. Cra20, drought stress, rhizosphere bacterial community, root architecture

## Abstract

The rhizosphere is colonized by a mass of microbes, including bacteria capable of promoting plant growth that carry out complex interactions. Here, by using a sterile experimental system, we demonstrate that *Sphingomonas* sp. Cra20 promotes the growth of *Arabidopsis thaliana* by driving developmental plasticity in the roots, thus stimulating the growth of lateral roots and root hairs. By investigating the growth dynamics of *A. thaliana* in soil with different water-content, we demonstrate that Cra20 increases the growth rate of plants, but does not change the time of reproductive transition under well-water condition. The results further show that the application of Cra20 changes the rhizosphere indigenous bacterial community, which may be due to the change in root structure. Our findings provide new insights into the complex mechanisms of plant and bacterial interactions. The ability to promote the growth of plants under water-deficit can contribute to the development of sustainable agriculture.

## Introduction

Drought is one of the key obstacles to improving crop growth and productivity in the world. It is estimated that drought has reduced national cereal productions by 9–10% (Naveed et al., 2014; Lesk et al., 2016). Drought periods affect plant water potential and enlargement, which can interfere with the normal changes of plant physiological and morphological characteristics (Hsiao, 2000; Vile et al., 2012). Vegetative phases and dry matter production are closely related to key developmental changes such as reproductive transformation (Jung and Muller, 2009; Wang et al., 2015b). Specifically, flowering time may change due to drought (Tisne et al., 2010; Bresson et al., 2013). Therefore, the development of drought-resistant cultivars and water-use-efficient plants has attracted worldwide attention (Lawlor, 2013). However, those traditional methods are technically demanding and labor-intensive, so are difficult to implement in practical applications (Niu et al., 2018).

In order to reduce the negative impact of water-deficit and complete the life cycle under adverse conditions, plants have developed several mechanisms to cope with drought stress (Farooq et al., 2009; Ngumbi and Kloepper, 2016). At the morphological level, the stems and roots are the most affected parts and are the main components of plants responding to drought. Plants generally limit the number and area of leaves and/or change the growth and development of the root system, root density and depth in response to drought stress (Schuppler et al., 1998; Kavar et al., 2007; Bresson et al., 2013; Zhan et al., 2015). Physiological changes occur quickly after the onset of water-deficit; for example, a rapid adjustment of the osmotic potential by accumulation of soluble substances helps maintain the turgor of the cells while increasing the driving force of water flow into the cells (Anjum et al., 2017).

Millions of microbes inhabit plant root systems, and the metabolic activities of microorganisms and their interactions with plants affect plant growth and productivity (Yang et al., 2009; Schmidt et al., 2014). Among those organisms, PGPRs can interact with numerous host plants and improve plant growth and health via various mechanisms, such as nitrogen fixation (Ferguson and Mathesius, 2014), P-solubilizing (Bashan et al., 2013), and competition with pathogens (Lee et al., 2015). Some PGPRs produce phytohormones or mediate plant signals by inducing plants to produce phytohormones (Zamioudis et al., 2013; Wang et al., 2015). Others can increase plant antioxidant enzyme content and activity, such as SOD, catalase (CAT), and POD, to alleviate oxidative damage caused by drought (Helena and Carvalho, 2008; Hasanuzzaman et al., 2014; Kaushal and Wani, 2016a).

Many PGPRs such as *Burkholderia phytofirmans*, *Pseudomonas fluorescens*, *Stenotrophomonas*, and *Bacillus amyloliquefaciens* are well known for their plant growth promoting effects and are used for improving crop plant performance under stress conditions (Fernandez et al., 2012; Zhou et al., 2016; Kim et al., 2017; Zhang et al., 2017). Do PGPRs affect the composition of plant root exudates, and change the indigenous rhizosphere microbial community? Possibly, as different strains have different effects and mechanisms. For example, inoculation with *B. amyloliquefaciens* could alter the indigenous rhizosphere microbial community of lettuce, but in the same experiment two other strains of *Bacillus*, *B. cereus*, and *B. subtilis* did not affect the lettuce rhizosphere dominant indigenous microbial community (Gadhave et al., 2018). Unfortunately, so far, the research on the effects of plant growth-promoting bacteria on plant indigenous microbial communities has been mainly focused on the genera *Bacillus*, *Pseudomonas*, and *Stenotrophomonas*, and almost no other genera have been analyzed in depth.

Many results show that PGPRs affect the overall plant phenotype and some physiological characteristics, but their detailed effects on plant growth, development, physiology of plants and the rhizosphere bacterial community under drought have not been well explored. Furthermore, the emphasis on physiology and hormone studies cannot fully explain the effects of growth-promoting bacteria on plants under drought conditions (Maxton et al., 2018). Moreover, most studies focus on a single time point, failing to explain drought’s effect on the dynamics of plant development, and reports on plant growth throughout the whole plant life cycle are lacking (Bresson et al., 2013).

*Sphingomonas* are well studied for their ability to degrade organic pollutants (Kunihiro et al., 2013; Niharika et al., 2013). However, recent studies have shown that *Sphingomonas* have a role in promoting plant growth (Khan et al., 2014; Pan et al., 2016). Cra20 was isolated from the root surface of *Leontopodium leontopodioides*, and belongs to the genus *Sphingomonas* of α-Proteobacteria. *In vitro* studies show that Cra20 enhances shoot and root growth of *A. thaliana*. However, the information about plant–bacteria interactions under soil conditions and the responses of plants under drought stress has not been investigated. Here, we study the growth dynamics, morphological and physiological changes of *A. thaliana* and the impact on rhizosphere indigenous microbial communities of *A. thaliana* under different water conditions. Therefore, our objectives were to (1) study the effects on the root architecture of *A. thaliana* inoculated with Cra20 *in vitro*; (2) study the long-term effects of Cra20 on *A. thaliana* growth using a non-destructive photographic technique; (3) analyze the impact on the rhizosphere indigenous bacterial community of *A. thaliana*; and (4) assess the effect of Cra20 on long-term *A. thaliana* development.

## Materials and Methods

### Cultivation of Rhizobacteria and Inoculated Treatments

*Sphingomonas* sp. Cra20 was isolated from the root surface of *L. leontopodioides* in the Tianshan Mountains, China. To confirm the isolated bacteria, PCR reactions were performed with the universal 16S primers 27f (5^′^-AGAGTTTGATCCTGGCTCAG-3^′^) and 1492r (5^′^-TACGGTTACCTTGTTACGACTT-3^′^). The PCR products were sequenced by BGI (Shenzhen, China) and the resultant 16S rRNA sequences were compared to GenBank database using BLAST. Cra20 was identified as a species of *Sphingomonas* with GenBank accession number JQ977105. To obtain the bacterial suspension, *Sphingomonas* sp. Cra20 was cultured on sterile R2A agar plates at 28°C. After 48 h of growth, a single colony was picked out and cultured in R2A liquid medium on a rotary shaker (140 rpm) at 28°C. After 36 h of growth, the culture of bacteria cells was pelleted by centrifugation (5000 rpm for 10 min, 20°C) and resuspended in sterile water. The bacterial titer was adjusted to an optical density at 600 nm of 0.2 (corresponding to 2 × 10^8^ CFU mL^-1^). To obtain 1 × 10^7^ CFU g^-1^ of soil, the inoculum was placed directly into the soil substrate, then manually homogenized.

### Plant Material and Growth Conditions *in vitro*

The seeds of *A. thaliana* Col-0 were surface sterilized with 2% sodium hypochlorite solution for 15 min, washed five times with sterile water, and sown on 1/2× MS agar supplemented with 1% sucrose. After 2 days of stratification at 4°C, the petri dishes were transferred and positioned vertically in a growth chamber under a long day photoperiod (16 h of light at 24°C, with light intensity of 100 μmol.m^2^.s^-1^, and 8 h of dark at 22°C). 5 days after germination, we selected the seedlings showing uniform growth and moved them to a new 1/2× MS medium (with added 0.05% acid hydrolysed casein, Cra20 did not grow very well in MS medium, acid hydrolyzed casein provided necessary nutrients for strain Cra20), and it was kept at a density of eight seedlings per plate, three plates per treatment. To the experimental group we added 20 μL 2 × 10^8^ CFU mL^-1^ bacterial suspension or 20 μL distilled sterilized water (as the control) at a 4 cm distance from the root tip and continued vertical cultivation for 7 days.

### Plant Material, Growth Conditions, and Irrigation Treatments

*Arabidopsis thaliana* Col-0 was used as a typical model plant in this study. A total of 144 individual plants were studied (one plant per pot, 36 plants per treatment). 18 pots were in one tray, 2 trays per treatment (Supplementary Table 1). Five seeds were sown at the soil surface in 300 ml culture pots filled with a damped mixture of nutrition soil (Substrate, Pindstrup) and roseite (1: 3, v/v) inoculated with or without Cra20. Non-inoculated soil was previously damped with sterile water to avoid differences in initial soil humidity between this soil and inoculated soil. Aside from Cra20, no bacteria were directly inoculated into the experimental pots. Instead, the bacterial communities likely originated from bacteria initially present in the soil mixture, which was not sterilized beforehand, and from the growth chamber environment. The 144 pots were placed in the dark at 4°C for 2 days to ensure germination at the same time and were then transferred into the controllable growth chamber. Trays rotated to change position every 3 days to ensure that all trays were treated uniformly, and an exploratory analysis of all datasets at the conclusion of the experiment confirmed that there were no substantial between-tray differences in plant phenotypes within treatment groups (*t*-test, all *P* > 0.1). Pots were dampened with sprayed sterile water once a day until germination. The seedlings were cultivated under condition of 12 h light (100 μmol.m^2^.s^-1^ photosynthetic photon flux density) and 12 h dark with the temperatures at 24: 22°C (light: dark). After the first two cycles true leaves emerged, the uniform growth seedlings were maintained, and others were pulled out to one seedling per pot. Soil relative water content was maintained at 40% (0.4 g H_2_O g^-1^ dry soil) in the WW treatment and kept at 25% (0.25 g H_2_O g^-1^ dry soil) as the WD treatment. The weight of each pot was adjusted every 3 days with sterile water to maintain these two values of soil water content until plants began bolting.

### Measurement of Plant Root Architecture Traits *in vitro*

To measure the shoot fresh weight, seedlings were sectioned at the root-shoot junction, and twelve groups of excised shoots were immediately measured on an analytical balance (two shoots were used in each group). The number of emerged LRs of at least 20 seedlings was counted every day using a dissecting microscope (C-DSS230, Nikon, Japan) for 7 days. For RH measurements, digital images were obtained every day from the primary root segment for a total of 7 days, located 2 mm above the root tip, using a dissecting microscope and a magnification of 15×. Then RH length and density were quantified with ImageJ software (ImageJ 1.48u; Rasband, Bethesda, MD, United States). The number of emerged RH branches of at least 20 seedlings was counted by using digital images with a dissecting microscope and a magnification of 50×.

### Measurement of Plant Traits in Potted Plants

For rosette expansion measurement, the projected area of the rosette was determined every 3 days from semiautomated analysis of zenithal images of the plants (SONY camera, Japan). The rosette leaf diameter of plants was measured by using a ruler, the length and width of every rosette were measured, then the average value of length and width was used as rosette leaf diameter. The number of leaves per plant visible to the naked eye was counted every 2–3 days or 1–2 days to determine the phyllochron in early vegetative growth phase and the later phases, respectively. That is, the time between having a new leaf visible to the naked eye until emergence of the bolting stem. The bolting time was determined as the number of days from germination to macroscopic visualization of flower buds (Boyes et al., 2001). Rosettes were cut from the roots and immediately weighed to determine shoot fresh weight. After the total leaf numbers were determined, the rosettes were then oven-dried at 70°C under constant weight to measure shoot dry weight. Roots were carefully separated from the soil, gently washed with deionized water to remove the attached soil, then placed in a dish at 70°C under constant weight to determine root dry weight.

To measure physiological changes, Chlorophyll a and b were extracted with acetone (80%) and the concentrations were determined by spectrophotometry, according to the absorbance coefficients determined by Wellburn (1994). Free proline content was determined using the acid ninhydrin method of Ait and Audran (1997). MDA was monitored by analyzing the concentration of thiobarbituric acid-reactive substances according to Ait et al. (2000). Activities of antioxidant enzymes were determined according to Jin et al. (2008). POD activity was determined based on the oxidation of guaiacol using H_2_O_2_ and measuring absorbance at 470 nm. SOD activity was determined by the photochemical method. One unit of SOD activity was defined as the amount of enzyme required to cause a 50% inhibition of the reduction of nitroblue tetrazolium, measured by absorbance at 560 nm, and the SOD specific activity was the activity of SOD per mg protein.

### Total Bacterial DNA Extraction, Polymerase Chain Reaction (PCR), and High-Throughput Sequencing of 16S rRNA Amplicons

We measured plant characteristics during the *Arabidopsis* bolting period and collected rhizospheric soil samples. The control samples of inoculated and non-inoculated soil were collected at 10 day after treatment. The rhizospheric soil samples of WND, WNW, WBW, and WBD were collected at 60, 62, 63, and 65 day after planting, respectively. Rhizospheric soil was collected according to an established method (Lundberg et al., 2012). Briefly, the whole root system was extracted from the pots, then slightly shaken to remove loosely adhering soil, and subsequently the whole root system was transferred to a 15 ml sterile tube. The tubes were vigorously shaken to collect rhizospheric soil, then the roots were removed. The rhizospheric soil samples from three plants were mixed together as a biological replicate, and three biological replicates were obtained per treatment (nine plants randomly selected from two trays). For the bulk soil, approximately 2 g of soil was collected from each control pot and transferred to a 15 ml sterile tube; similarly to the sampling method for rhizospheric soil, the bulk soil from three pots was collected as a biological replicate, with three biological replicates being obtained per treatment. All samples were stored at -20°C for further DNA extraction.

The total bacterial DNA extraction, PCR and High-Throughput Sequencing of 16S rRNA Amplicons were carried out by Sagene Biotech (Guangzhou, China) using an Illumina MiSeq (PE300). Briefly, DNA was extracted from the soil samples (0.5 g) using an E.Z.N.A.^TM^ Soil DNA Kit (Omege, Bio-Tek Inc., Norcross, GA, United States) according to the protocol of the manufacturer. The quantity and quality of the DNA extracts were determined using a NanoDrop 1000 spectrophotometer (Thermo Fisher Scientific Inc., Waltham, MA, United States). Then the extracted DNA was stored at -20°C for future analyses. An aliquot of the extracted DNA from each sample was used as the template for amplification. Partial 16S rRNA genes were amplified targeting the variable V3-V4 regions and using primers 341F (5^′^-CCTAYGGGRBGCASCAG-3^′^) and 806R (5^′^-GGACTACNNGGGTATCTAAT-3^′^). The PCR reactions were performed in a 50 μL mixture containing 1.0 μL template DNA (20 ng/μL), 1.0 μL of each primer at 5 μM, 10 μL of 5 × PrimeSTAR buffer, 4 μL of deoxyribonucleoside triphosphate (dNTP) at 2.5 mM, 0.5 μL of PrimeSTAR HS DNA Polymerase (2.5 U/μL, Takara Bio, Dalian, China), and 32.5 μL of ultrapure sterile water. The following thermal program was used for amplification: 98°C for 1 min, followed by 27 cycles of denaturation at 98°C for 30 s, annealing at 55°C for 30 s, extension at 72°C for 30 s, and a final extension at 72°C for 5 min. The PCR products were purified using AMPure XP beads (Agincourt, Beckman Coulter, Beverly, MA, United States). Subsequently, library quantification, normalization and pooling were performed and MiSeq v3 reagent kits were used to finally load the samples for MiSeq sequencing. Sequences were converted to FASTA format and concatenated into a single file. All reads were clustered into OTUs with 97% similarity cutoff using UPARSE (version 7.1^1^) and chimeric sequences were identified and removed using Trimmomatic software. The taxonomy of each 16S rRNA gene sequence was analyzed by the RDP Classifier^2^ against the SILVA (SSU115) 16S rRNA database using a confidence threshold of 70% (Amato et al., 2013). Finally, a filtered OTU table was obtained for further analysis. The raw metagenome read data are deposited in the National Center for Biotechnology Information (NCBI) Short Read Archive (BioProject ID: PRJNA532738^3^).

The Shannon-Wiener and Simpson indices were used to evaluate the microbial community diversity, and the Chao1 estimator (Chao1) and abundance based coverage estimator (Ace) indices were used for the microbial community richness estimates at the OTU level. One-way ANOVA and Tukey HSD were performed in RStudio. For the Beta-diversity calculations, the whole filtered OTU table was used and normalized using the function cumNorm from the R package metagenomeSeq (v.1.12) (Paulson et al., 2016). Hierarchical clustering analysis was performed using Bray–Curtis distance and visualized using treeview. The figures were generated with SigmaPlot 11.0 and Excel. In order to investigate the overall differences in community composition among the samples, principal coordinate analysis was performed using Bray-Curtis distance (Vermeesch et al., 2016), and PERMANOVA was used to analysize the effect of Cra20, water and both treatment on the rhizosphere community by the function adonis from the R package vegan (v.1.12) with 9,999 permutations (Narrowe et al., 2015). The linear discriminant analysis (LDA) effect size (LEfSe) analysis was performed by using the website http://huttenhower.sph.harvard.edu/LEfSe. The non-parametric factorial Kruskal–Wallis rank-sum test with the *p*-value < 0.05 was used to detect features with significantly different abundances at OTU level between groups.

### Statistical Analyses

Statistical analysis was performed using the SPSS statistical 19.0 software (SPSS Inc., United States). Data were tested at a significant level of *P* < 0.05 using Student’ *t*-test and one-way ANOVA, Graphical work was performed using OriginPro 9.0 (Northampton, United States).

## Results

### Cra20 Regulated the Development of *A. thaliana* Root System Architecture

**FIGURE 1:**
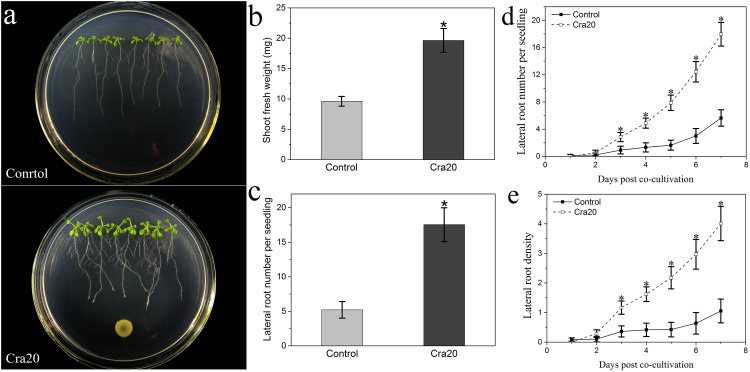
Effects of *Sphingomonas* sp. Cra20 inoculation on the growth of *A. thaliana* Col-0 seedlings. **(a)** Representative images of seedlings growing on control plates and plates containing Cra20. **(b)** Shoot biomass production measured after 7 days of co-culture with Cra20. **(c)** Lateral root number per seedling measured after 7 days of co-culture with or without Cra20. **(d)** Time course of lateral root number in response to Cra20. (e) Time course of lateral root density in response to Cra20. Data represent mean fresh weights ± SD of twelve groups of seedlings each consisting of two excised shoots. Lateral root number ± SD of at least 20 seedlings from three different plates. Asterisks indicate statistically significant difference compared with Control-treated roots (Student’s t-test; P < 0.001). The experiment was repeated three times with similar results.

The plant growth promoting effect of strain Cra20 was investigated on *A. thaliana* Col-0 seedlings growing vertically on agar-solidified medium. After 7 days of co-culture, it was found that the strain Cra20 was capable of stimulating *A. thaliana* seedlings biomass production (Figure 1a), and increased the shoot fresh weight of seedlings by 2.1 times (Figure 1b). In addition to stimulating shoot fresh weight, the strain Cra20 triggered a number of developmental alterations.

**FIGURE 2:**
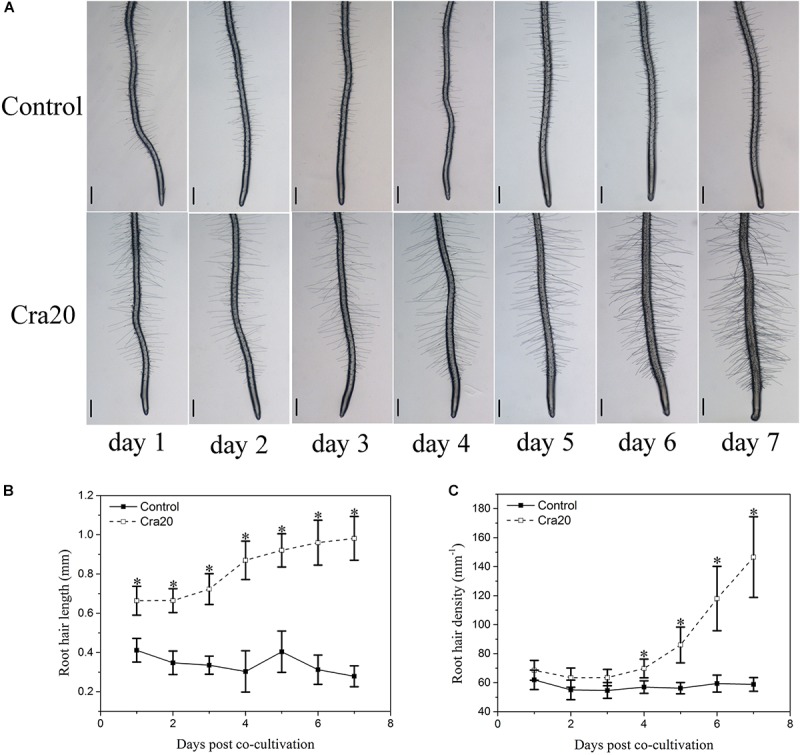
Effects of *Sphingomonas* sp. Cra20 on *A. thaliana* root hair development. **(A)** Representative images of *A. thaliana* root tip showing root hair formation after 7 days of growth on control or with Cra20 plates. The scale bars represent 200 μm. **(B)** Time course of root hair length in response to Cra20 (20 root hairs per plant, 8 plants from three different plates) and **(C)** Time course of root hair density in response to Cra20 in the root segment located 2 mm above the root tip (eight plants from three different plates) of *Arabidopsis* seedlings growth on control or with Cra20 plates. Asterisk indicate statistically significant differences compared with control treated roots (Student’s *t*-test; *P* < 0.001). The experiment was repeated three times with similar results.

One of the most prominent Cra20-mediated morphological alterations in the root system architecture was the stimulation of LR formation (Figure 1a). We measured a 3.4 times increase in the number of emerged LRs on Cra20-treated roots compared with control roots after 7 days of co-culture (Figure 1c). Furthermore, the time course experiment showed that there was no difference between control and inoculated treatment at the first day. But the formation rate of LR inoculated with Cra20 was increased from the second day, and the LR number was significantly higher than control after 3 days of co-culture (Figure 1d). Similarly, the LR density increased rapidly in Cra20-treated seedlings, but only slightly in control plants (Figure 1e). In addition to positive effects on LR formation, Cra20 has a strong impact on RH development (Figure 2A). In the course of the experiment, the results showed that the RH length of the control plants did not change with the culture time, while the inoculation of Cra20 promoted the elongation of RHs, even on the first day of co-culture, and increased rapidly with the culture time (Figure 2B). Otherwise, the RH density was not changed when inoculated with Cra20 in the first 3 days, but the RH density increased rapidly in Cra20-treated seedlings after third day of co-culture. In particular, after 7 days of co-culture, there was a more than 2-fold increase in the RH density of Cra20 exposed plants (Figure 2C). To test any effect mediated through soluble or volatile organic compounds (VOCs) produced by Cra20, plastic petri dishes that contained a center partition were used. It was found that Cra20 promoted the growth of *A. thaliana* by producing VOCs, and induced the formation of LRs (Supplementary Figure 1).

### Effects of Strain Cra20 on Lateral Branches of *A. thaliana* Root Hair

**FIGURE 3:**
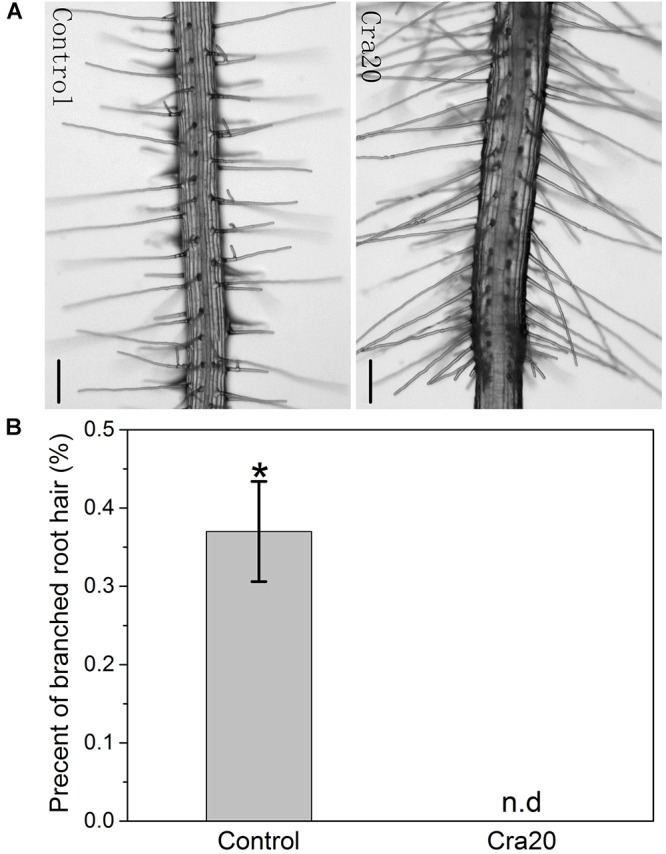
Inoculation of *Sphingomonas* sp. Cra20 affected *A. thaliana* development of branched root hair. **(A)** Images of representative 12-day-old *A. thaliana* branched root hairs after 7 days of growth under control and Cra20-inoculated conditions. The scale bars represent 100 μm. **(B)** Effects of Cra20 inoculated on percent of branched root hair per seedling. Data are mean ± SD of three independent experiments (15 root hairs per plant, 6 plants from three different plates). Asterisk indicate statistically significant differences compared with control treated roots (Student’s *t*-test; *P* < 0.001). The experiments were repeated three times with similar results.

Five-day-old *A. thaliana* seedlings were transferred to new 1/2 MS agar medium supplemented with 1% sucrose and 0.5% Casein acid Hydrolysate, and the new medium were supplemented with Cra20 or not. After 7 days, we found that about 36% of the RHs formed lateral branches in the control group (arrows in Figure 3A), while the RHs co-cultured with Cra20 did not form branches (Figure 3B). The formation of RH branches can be conducive to plant adaptation to drought (Bobrownyzky, 2016). We believe that the lack of this action in Cra20 is more than overcame by other properties which give it the ability to improve drought tolerance in *Arabidopsis*.

### Cra20 Promoted Growth of *A. thaliana* Col-0 Under Both Water Content Condition

**FIGURE 4:**
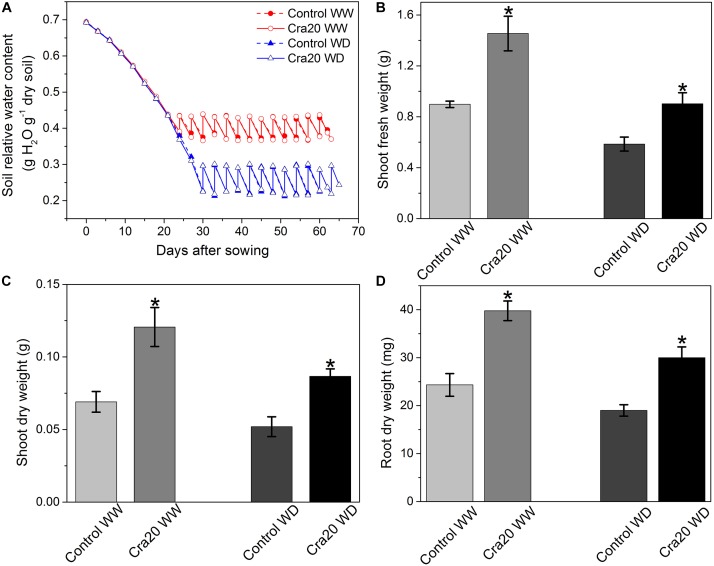
Effects of *Sphingomonas* sp. Cra20 and water-deficit on above- and below-ground mass of *A. thaliana*. **(A)** Time course of relative soil water content mean during plant growth. **(B)** Shoot fresh weight. **(C)** Shoot dry weight. **(D)** Root dry weight; of inoculated with Cra20 and non-inoculated (control) plant under well-watered (WW) and water-deficit (WD) conditions measured at bolting. Data are means ± SD of nine plants. Asterisk indicate statistically significant differences compared with control treated roots (Student’s *t*-test; *P* < 0.01).

We observed that Cra20 could promote the development of *A. thaliana* root structure, which contributes to the adaptability of plants in an arid environment (Bao et al., 2014). We thought that Cra20 may have the ability to improve plant drought resistance. Research indicated Cra20 had a growth-promoting effect on the *A. thaliana* under both the WW and WD soil condition. The time course of relative soil water content during plant growth is shown in Figure 4A. Under WW, soil inoculation induced a 61.97% increase of above-ground vegetative fresh weight at emergence of the flowering buds (bolting stage) (Figure 4B) and induced a 74.63% increase in shoot dry weight (Figure 4C). Plant root dry weight increased by 63.51% after inoculation with Cra20 (Figure 4D). Under WD, there was also a great increase after inoculation of Cra20. The shoot fresh weight and root dry weight both increased by about half, reaching 54.05 and 57.89%, respectively (Figure 4B,D), while plant shoot dry weight had a 66.74% increased (Figure 4C). Furthermore, the shoot fresh weight of the non-inoculated plant under WW was basically the same as the above-ground fresh weight of the plant inoculated with Cra20 under WD (Figure 4B). Conversely, the plant inoculated with Cra20 under WD had more dry weight both above- and below-ground than the non-inoculated plant under WW (Figure 4C,D). Although the increase in shoot fresh and dry weight under WD conditions was lower than WW, the shoot dry matter content was almost the same (Supplementary Figure 2).

### The PGPR Strain Cra20 Induced Changes in the Developmental Dynamics of *A. thaliana* Col-0 Plants

**FIGURE 5:**
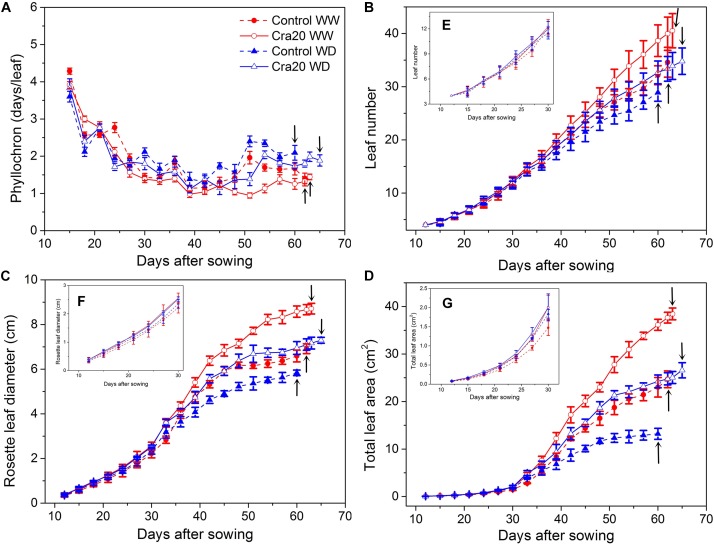
Effects of *Sphingomonas* sp. Cra20 and water-deficit (WD) on growth and development dynamics of *A. thaliana* Col-0. **(A)** Phyllochron; **(B)** leaf number; **(C)** rosette leaf diameter; **(D)** total leaf area of control and inoculated with Cra20 under well-watered (WW) and WD conditions. Insert **(E)** shows the leaf number in the first 30 days after sowing. Insert **(F)** shows the rosette leaf diameter in the first 30 days after sowing. Insert **(G)** shows the total leaf area in the first 30 days after sowing. Arrows indicate bolting time. Data are means ± SD of 30 plants (Student’s *t*-test; *P* < 0.001), the phyllochron, leaf numbers, rosette leaf diameter, and total leaf area are estimated from 3 day intervals around each time point.

The effects of soil inoculated by Cra20 on the growth and development of *A. thaliana* appeared later than when it was cultured *in vitro*. The number of leaves increased faster after the two euphylla grew, but the growth rate of the leaves slowed down in the late period of vegetative growth. We observed that *A. thaliana* inoculated with Cra20 had a smaller phyllochron, under both WW and WD conditions (Figure 5A). However, this was not due to the different germination potentials of inoculated and non-inoculated plants, the leaves appeared in basically the same time for both the first two cotyledons and the two euphylla (Figure 5B,E). At the beginning of the drought treatment on the 24th day, the phyllochron began to differ between WW and WD treatments. The growth of *A. thaliana* was significantly affected under drought treatment, especially the non-inoculated plants (Figure 5A).

Rosettes of *A. thaliana* inoculated with Cra20 had a larger diameter than non-inoculated plants after two euphylla appeared; there are significant differences between inoculated and non-inoculated plants in both WW and WD treatment groups. However, rosette diameter changes were not obvious in the first 30 days under all conditions (Figure 5F). After 9 days of drought treatment, the relative expansion rates of rosettes were significantly reduced, whether they were inoculated or not (Figure 5C, *P* < 0.001). Although the inoculated treatment maintained a fairly high relative expansion rate, it did not lead to an earlier bolting of the treated plants. In contrast, the inoculated and non-inoculated plants differed only by 1 day under WW conditions (*P* = 0.45), and on average, were delayed by 5 days under WD conditions (*P* < 0.05) (Figure 5B and Supplementary Figure 3). Moreover, the inoculated plants produced more leaves at bolting in both water conditions (Figure 5B, *P* < 0.001), although there were no significant changes between inoculated and non-inoculated plants during the first 30 days under both WW and WD (Figure 5E). As the bolting time was related to the duration of vegetative growth in *A. thaliana*, it was not surprising to observe that inoculated plants exhibited a larger rosette area expansion, especially during bolting in both water conditions (Figure 5D and Supplementary Figure 4, *P* < 0.001). Total leaf area of *A. thaliana* inoculated with Cra20 increased by 55.7% over the non-inoculated under WW, while total leaf area was two times that of the non-inoculated under WD (Figure 5D). However, total leaf area changes were not obvious in the first 30 days under all conditions (Figure 5G). The larger effect of Cra20 under WD compared with WW resulted in a better tolerance to WD.

### Cra20 Affected Whole Plant Physiology and Antioxidative Enzymes of *A. thaliana*

**FIGURE 6:**
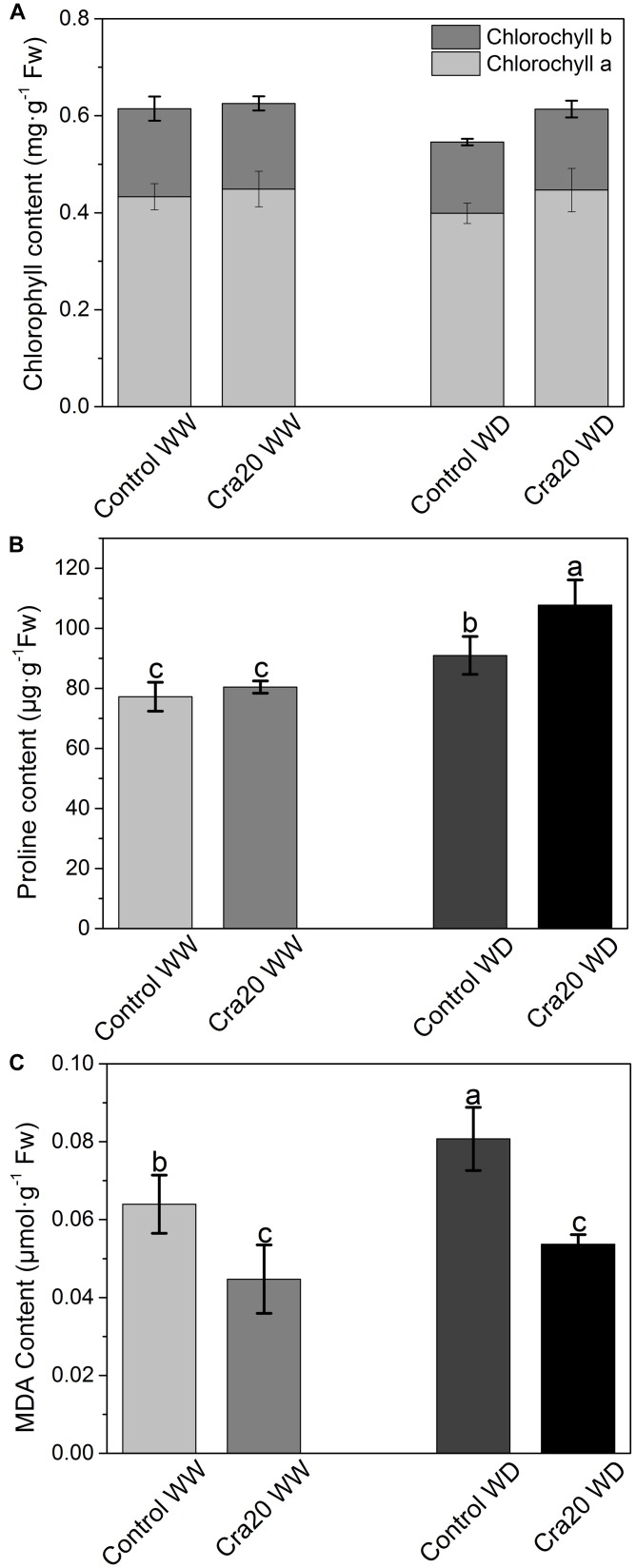
Effects of *Sphingomonas* sp. Cra20 and water-deficit on Physiological change of *A. thaliana* Col-0. **(A)** Chlorophyll a and b content of shoot. **(B)** Proline content. **(C)** MDA content of inoculated with Cra20 and non-inoculated (control) plant under well-watered (WW) and water deficit (WD) conditions measured at bolting. Data are mean ± SD of five independent experiments (leaves from six to eight plants). Different letters indicate statistically significant differences (ANOVA Duncan test; *P* < 0.05).

We measured whole plant physiology and antioxidative enzymes of *A. thaliana* at the bolting time. Chlorophyll content was not affected by either the amount of water or the presence of the bacteria in the soil (Figure 6A). However, the content of chlorophyll a and b in non-inoculated under water deficit was slightly lower (Figure 6A). There was no difference of proline content between inoculated and non-inoculated plants under WW (Figure 6B), while *A. thaliana* inoculated with Cra20 has higher proline under WD (Figure 6B). Our studies showed that inoculation reduced the cumulative amount of MDA and reduced it by 33% under WD (Figure 6C). These results indicated that Cra20 was capable of increasing the tolerance of *A. thaliana* to drought.

**FIGURE 7:**
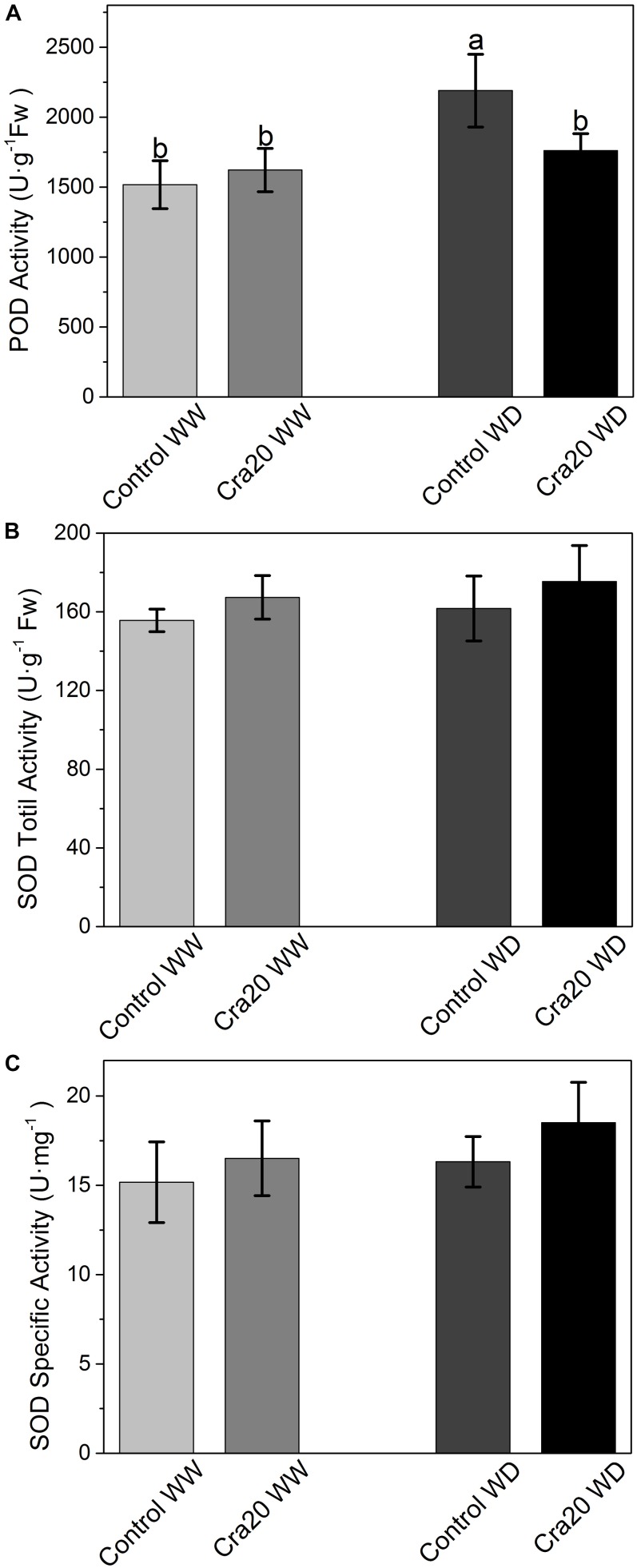
Effects of *Sphingomonas* sp. Cra20 and water-deficit on antioxidative enzymes change of *A. thaliana* Col-0. **(A)** POD activity. **(B)** SOD total activity. **(C)** SOD specific activity of inoculated with Cra20 and non-inoculated (control) plant under well-watered (WW) and water-deficit (WD) conditions measured at bolting. Data are mean ± SD of five independent experiments (leaves from six to eight plants). Different letters indicate statistically significant differences (ANOVA Duncan test; *P* < 0.05).

To investigate whether drought and inoculation caused changes in the antioxidant enzyme system, the POD and SOD activities were determined. We found that water deficit can cause an increase in POD activity without inoculation (Figure 7A), however, there was no significant difference between inoculated and un-inoculated plants in the two different water content conditions, finding some un-inoculated plants with higher POD activity than inoculated ones under WD (Figure 7A). SOD total activity and SOD specific activity increased slightly after inoculation, but there was no significant change (Figure 7B,C).

### The Effect of Cra20 on the Rhizosphere Bacterial Community of *A. thaliana* in Pot Experiments

**FIGURE 8:**
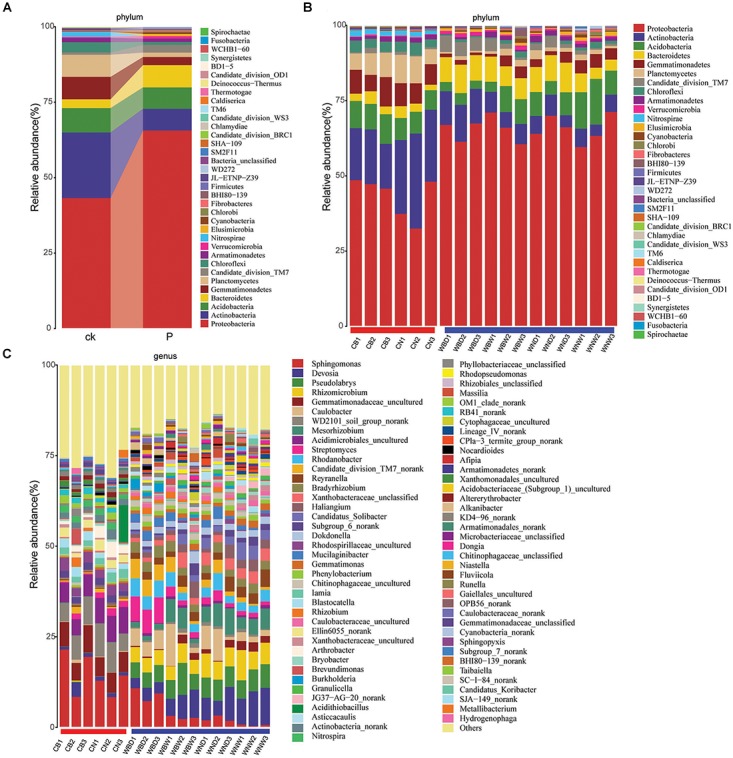
Relative abundance of soil and rhizosphere bacterial community. The notations ck., P represent control soil and plants rhizosphere, respectively. **(A)** Merged phylum; **(B)** phylum; and **(C)** genus.

The rhizosphere bacterial community composition was analyzed, and after quality trimming and chimera removal, the bacterial sequences were clustered from 1476 to 4782 OTUs using a Bayesian classifier at the 97% similarity level in different samples. Global Alignment for Sequence Taxonomy was used for taxonomic assignment of 16S rRNA sequences, and a total of 35 phyla were identified by pooling sequences from all samples. Among them, Proteobacteria, Actinobacteria, Acidobacteria, Bacteroidetes, Gemmatimonadetes, Planctomycetes, and Saccharibacteria (also known as TM7) were the dominant bacteria in all samples. Proteobacteria was the most dominant phylum in all samples. Actinobacteria has a high proportion in the control group, but relatively few in the rhizosphere of *A. thaliana*. Furthermore, the Bacteroidetes and TM7 were dominant phyla in the rhizosphere compared to controls, while the Gemmatimonadetes, Planctomycetes and Chloroflexi were more dominant phyla in the control groups (Figure 8A). Otherwise, the Actinobacteria and Bacteroidetes were more abundant, but Acidobacteria and Gemmatimonadetes were relatively less abundant under drought treatment (Figure 8B).

It was also notable at the genus level that *Sphingomonas* was the most abundant genus, especially in CB groups. But we found that the content of *Sphingomonas* was decreased, possibly due to the presence of plants, and was replaced by *Devosia*, *Pseudolabrys*, and other genera (Figure 8C). On the other hand, it was found that the proportion of *Sphingomonas* was still relatively high in the water-deficit (WBD) treatment groups, suggesting it played an important role in drought treatment, which might be the reason why Cra20 could improve drought resistance of *A. thaliana* (Figure 8C).

The bacterial community diversity between the controls and the rhizosphere was significantly different, but there was no difference between the different treatments (Table 1). In the control group, the soil bacterial community diversity and abundance were higher whether it was inoculated or not inoculated (Supplementary Figure 5). That indicated plants have a selective effect on rhizosphere bacteria and inhibit bacterial diversity.

**Table 1:** Soil and rhizosphere bacterial diversity and abundance among different treatments.

Samples	ACE	Chao1	Shannon	Simpson (10^-2^)
CN	1674.87 ± 80.40 a	1696.32 ± 92.67 a	5.83 ± 0.29 a	1.08 ± 0.27
CB	1622.56 ± 88.84 a	1647.44 ± 68.81 a	5.78 ± 0.15 a	1.01 ± 0.36
WNW	989.67 ± 36.23 b	997.99 ± 49.28 b	5.16 ± 0.09 b	1.58 ± 0.28
WND	1045.82 ± 34.58 b	1068.05 ± 61.26 b	5.10 ± 0.08 b	1.57 ± 0.21
WBW	1048.16 ± 47.54 b	1082.38 ± 65.78 b	5.17 ± 0.19 b	1.51 ± 0.21
WBD	1011.87 ± 67.95 b	1034.96 ± 80.89 b	5.10 ± 0.13 b	1.60 ± 0.29
*F*	79.499	62.574	12.552	1.917
*P*	0.000	0.000	0.000	0.165


*Different letters indicated significant differences following the ANOVA Duncan test at *P* < 0.05.*

**FIGURE 9:**
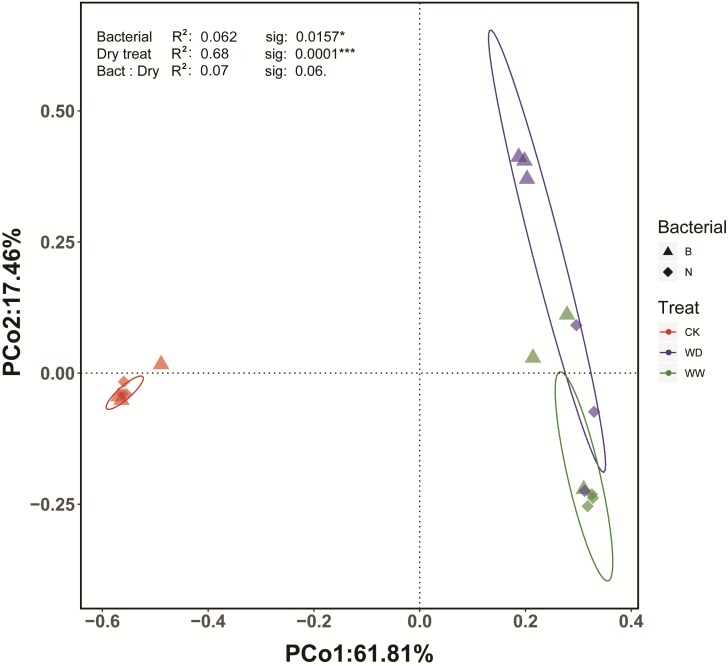
PCoA plot representing the Bray–Curtis beta diversity distance matrix across different treatments.

Beta diversity was portrayed using the Bray-Curtis distance measure (Lozupone et al., 2007). The Principal coordinates analysis (PCoA) indicated that the first two principal coordinates accounted for 61.8 and 17.5% of the variation within the matrix, respectively. That formed a very good representation of that matrix, with the first two principal coordinates accounting for almost 80% of the variation in the Bray-Curtis distance matrix. The result demonstrated there were differences in the bacterial community between control and plant rhizosphere groups (Figure 9). PERMANOVA analysis showed significant effects of both Cra20 treatment (*F* = 4.3, *P* < 0.05) and water treatment (*F* = 22.3, *P* < 0.001), with no interaction effect between the two factors (*F* = 2.3, *P* = 0.06), indicating that Cra20 inoculation could lead to statistically significant differences in the rhizosphere community composition. Because our analyses relied on relative abundance measurements, a significant yet misleading statistical effect of Cra20 treatment on bacterial community composition could potentially arise simply from Cra20 itself growing to a high abundance. This could inflate the relative abundance of *Sphingomonas* and decrease the relative abundance of other taxa, even if Cra20 inoculation had no effect on the absolute abundance of the other taxa in the community. In order to explore this problem, we performed PERMANOVA analysis with all *Sphingomonas* reads removed. The effect of Cra20 inoculation on rhizosphere community composition persisted in this analysis (*P* < 0.05), confirming that Cra20 inoculation alters community composition through its effects on the abundance of other microbial taxa (Supplementary Table 2).

**FIGURE 10:**
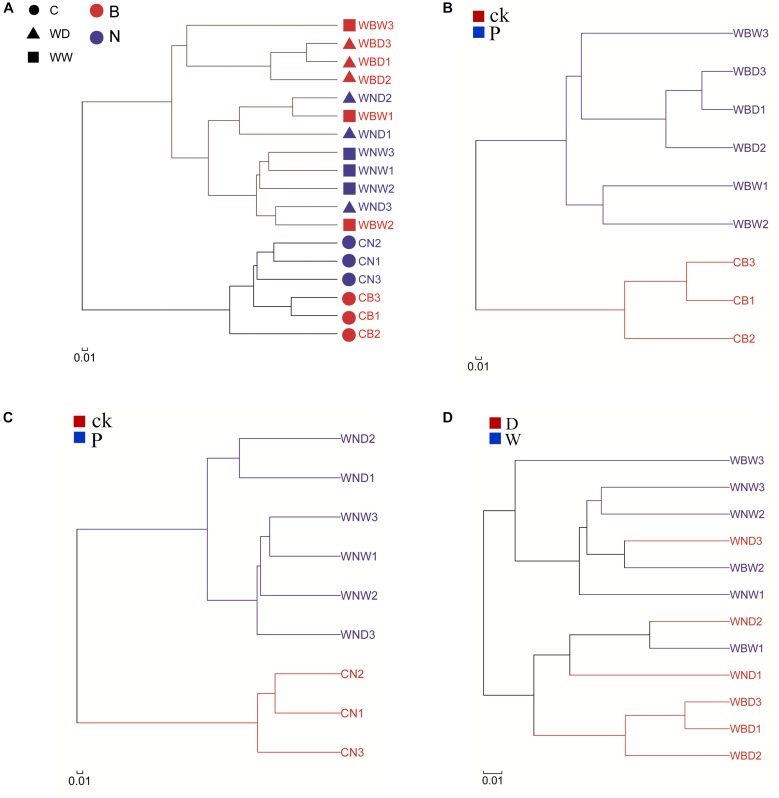
Hierarchical clustering tree of the bacterial community composition at the OTU level based on Bray–Curtis distances. **(A)** All samples; **(B)** inoculation; **(C)** non-inoculation; **(D)** comparison of the rhizosphere bacterial community without control soil.

A cluster analysis was used to analyze the similarity of the bacterial community composition among the different treatments at the OTU level (Figure 10A). The results showed that the control groups and plant rhizophere groups were on two different branches, indicating that there were differences in bacterial community between them, which might be due to selection by plants. At the same time, we found that the CB and the CN were also in two different branches, suggesting that inoculating with Cra20 affected the bacterial community composition (Figure 10A). Plants have a great effect on the selection of bacteria, so adding external bacteria could only temporarily change the rhizosphere indigenous bacterial community (Qiao et al., 2017). This indicates that plants have a recovery effect on the indigenous bacterial community. However, we also found that there was still a difference between the inoculation treatment and the non-inoculation treatment, especially in the bacterial community under the water-deficit conditions (Figure 10A). To determine the effects of water-deficit and inoculation on bacterial community structure, we performed cluster analysis on the inoculation treatments alone. The results showed that the control (CB), water-deficit (WBD), and well-watered (WBW) treatment were in three different branches, indicating that water-deficit affected the rhizosphere indigenous bacterial community under the conditions of inoculation (Figure 10B). To determine whether water-deficit had the same effect without inoculation, we carried out cluster analysis on non-inoculation treatments. The results showed that although there were overlaps between water-deficit (WND) and well-watered (WNW) treatments, there were still differences, indicating that water-deficit can affect the rhizosphere indigenous bacterial community without inoculation (Figure 10C). Comparing inoculation and non-inoculation treatments, we found that there was difference. WBD and WNW treatments were different, but in the case of WBW and WND treatments, bacterial community composition was more similar, probably because the treatments provided a similar effect on the bacterial community, and there was a more pronounced change when both conditions were applied simultaneously (Figure 10D). In summary, both inoculation and WD treatment could affect the composition of the rhizosphere indigenous bacterial community. Inoculation with Cra20 increased the resistance of *A. thaliana* to water-deficit, which might be due to changes in the rhizosphere bacterial community.

**FIGURE 11:**
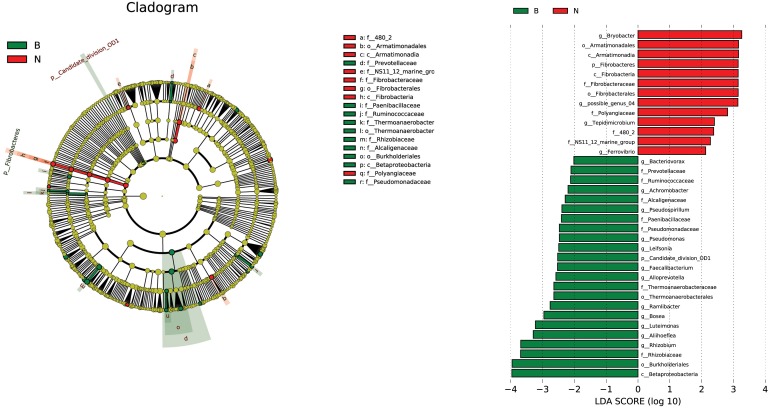
Linear discriminant analysis (LDA) effect size taxonomic cladogram highlighting the microbial biomarkers that statistically and biologically differentiated each group and depicting operational taxonomic units with absolute LDA score log10 > 2. B represents all the treatments inoculated with Cra20, N represents all the treatments of non-inoculation. Significant (red and green) and non-significant (yellow) discriminant taxonomic nodes are colored. The circle diameter is proportional to the taxon abundance, and each ring represents a taxonomic level in order from the center to the periphery: phylum, class, order, family, and genus.

The LEfSe algorithm for linear discriminative analysis (LDA) was used to compare all inoculated with non-inoculated groups to identify the species that were significantly different between the groups. The OTUs with the highest LDA (LDA log score threshold >2) from each group were depicted in Figure 11. Our results showed that the LEfSe algorithm detected that 23 differentially abundant taxonomic clades were sorted into the inoculation group (B) and 13 were found to belong to the non-inoculation group (N) (Figure 11). This may be due to the effects of inoculation with Cra20. The Betaproteobacteria and Burkholderiales were enriched in inoculation treatment, and *Bryobacter* was enriched in non-inoculation treatment. Otherwise, the genera *Rhizobium* and *pseudomonas* were enriched inoculation treatment, both these genera are generally considered as plant-growth promoting bacteria in some studies. In conclusion, inoculation with Cra20 caused changes in the rhizosphere indigenous bacterial community, which might have contributed to plant growth and drought resistance.

## Discussion

Plant growth-promoting rhizobacteria are associated with plant roots and increase plant productivity and resistance to abiotic stress. There is a large variety of mechanisms for the different kinds of PGPR (Friesen et al., 2011; Kaushal and Wani, 2016b). Here, we showed that *Sphingomonas* sp. Cra20 isolated from the root surface of *L. leontopodioides* promotes *A. thaliana* Col-0 growth and enhances *A. thaliana* tolerance to water-deficit. Moreover, our results show that inoculation with Cra20 impacted the rhizosphere indigenous bacterial community under water-deficit, and the *Sphingomonas* played an important role in the changes of rhizosphere bacterial community. This was the first time that the effect of *Sphingomonas* on the rhizosphere indigenous bacterial community has been analyzed; previous studies have mostly focused on *Bacillus* and *Pseudomonas* (Zhu et al., 2010; Chowdhury et al., 2013; Gadhave et al., 2018). Specifically, our results show for the first time that inoculation with PGPR does not affect the vegetative time under well-water condition, and allowed the accumulation of more biomass by increasing the growth rate of the plants, while there was a short delay in the bacterial treatment under water-deficit conditions.

### Cra20 Promoted *A. thaliana* Growth by Increasing the Lateral Root Number and Promoting the Formation of Root Hairs

Lateral roots and RHs constitute important features of the root structure, which promote plant anchoring in the soil and increases roots’ ability to absorb water and nutrients. Many PGPR strains are known to cause alterations in the root structure of plants, promoting the increase of LRs and the formation of RHs (Felten et al., 2009; Zamioudis et al., 2013; Wang et al., 2015). In this work, we used the newly isolated bacterium *Sphingomonas* sp. Cra20 as the inoculant to analyze the effects on *Arabidopsis thaliana*. Our results indicated that Cra20 improved the growth of *A. thaliana* (Figure 1), and altered the root structure by increasing the number of LRs and the formation of RHs (Figures 1, 2). More LRs and longer RHs will result in plants having a larger root surface in contact with the soil, thus helping plants absorb more water and nutrients through the whole root system (Bresson et al., 2013).

The promoting effect of PGPR on plants are mainly manifested in the production of phytohormones or induction of modifications in phytohormone signaling by VOCs (Jiang et al., 2012; Wang et al., 2015). It was also shown that Cra20 promoted the growth of *A. thaliana* by producing VOCs, and induced the formation of LRs (Supplementary Figure 1). 2, 3-butanediol and acetoin produced by *Bacillus subtilis* GB03 promote *A. thaliana* growth through phytohormone signaling (Ryu et al., 2003). Otherwise, 2, 3-butanediol produced by *Pseudomonas chlororaphis* O6 inductes systemic tolerance to drought in *A. thaliana* (Cho et al., 2012).

### Water-Deficit Induced the Formation of Root Hair Branches

Root hairs are tubular structures developed from epidermal cells called trichoblasts, which greatly increase the root surface area and help absorb water and nutrients (Carol and Dolan, 2006). RHs branch in some specific conditions, such as iron deficiency, decrease in reactive oxygen species or water-deficit (Mueller and Schmidt, 2004; Lohar et al., 2007; Bobrownyzky, 2016). The extracellular metabolite N-Acetylglutamic Acid produced by *Rhizobium trifolii* ANU843 can also induce the RHs to branch in white clover roots (Hollingsworth et al., 1991). The formation of RH branches under water-deficit helps to increase root surface area and absorption of water. Our results showed that a large number of RH branches were formed in the control group *in vitro*, which might be due to the lower amount of available water in petri dishes, resulting in a water-deficit condition (Figure 3). However, there were no RH branches under Cra20-treatment, probably because Cra20 instead promoted the roots to absorb more water by increasing the length of RHs (Figures 2, 3). Ammonium can stimulate the formation of RH branches, while the effects can be suppressed by ethylene in *A. thaliana* (Yang et al., 2011), while ethylene can promote elongation of RHs (Galland et al., 2012; Liu et al., 2017). Considering that Cra20 was isolated from the Tianshan Mountains, a water-deficit environment, we hypothesized that Cra20 can improve the tolerance of plants to water-deficit.

### Cra20 Promoted Plant Biomass Accumulation by Increasing Plant Growth Rate Under Both Water Content Condition

The transition from vegetative growth to reproductive development is an important event that determines the production of plant biomass (Jung and Muller, 2009). Reproductive transformation can be influenced by various kinds of abiotic changes, such as water availability and light length (Cerdán and Chory, 2003; Bresson et al., 2013), and by biotic influences, such as PGPR, which result in an accelerated or decelerated progress toward the vegetative phases (Bresson et al., 2013; Wang et al., 2015b). Here, we studied the effect of inoculation with Cra20 on the growth of *A. thaliana* in soil and found that the growth dynamics were affected. Cra20 induced growth changes after two euphylla appeared. The phyllochron was reduced in inoculated plants, indicating that inoculation treatment accelerated the growth rate of plants. The vegetative time was not shortened, although the growth rate of the plants was increased. *A. thaliana* inoculated with *Pseudomonas* sp. show a faster growth rate and accumulate more biomass, but plants reach the reproductive stage earlier (Schwachtje et al., 2011). Switchgrass (*Panicum virgatum* L.) plants inoculated with *B. phytofirmans* strain PsJN, reached the reproductive stage earlier, but the aboveground biomass accumulates decreased slightly (Wang et al., 2015a). Another work has exhibited that inoculation with *Phyllobacterium brassicacearum* STM196 reduced the growth rate leading to delayed reproductive time, and caused it to accumulate more biomass due to more vegetative growth time (Bresson et al., 2013). This indicates that various PGPR strains mediate different plant responses. In addition, the current study was the one to find that PGPR accelerated the growth of plants but did not affect the vegetative growth time under well-water condition.

Water-deficit inhibits plant growth and induces plants to produce more reactive oxygen species to eliminate superoxide anions, hydroxyl radicals and hydrogen peroxide which harm cell membranes and lead to cell death (Anjum et al., 2017). Inoculation of PGPR can induce plants to produce more antioxidant systems to survive under drought stress (Sandhya et al., 2010). However, our results showed lower activity of antioxidant enzymes, mainly because the enhancement of root systems increased the absorption of water and eased stress caused by water-deficit. A similar result is found in wheat, indicating that PGPR improve plants’ homeostatic mechanisms due to priming (Kasim et al., 2013). Analysis of the complete genome of Cra20 showed that Cra20 contained multiple cold shock protein genes (data not shown), which are reported to enhance maize and wheat resistance to drought stress and improve grain yield (Castiglioni et al., 2008; Yu et al., 2017). In conclusion, we considered that Cra20 improved water uptake and utilization efficiency mainly through improving root development, thereby promoting plant growth under drought conditions.

### Cra20 Changed the Rhizosphere Indigenous Bacterial Community

The rhizosphere is one of the richest nutrition ecosystems on Earth due to plant roots exuding abundant amounts of the photosynthetically fixed carbon into the rhizosphere (Bais et al., 2006). Different plant root exudates may be quite different, resulting in a significantly difference in the rhizosphere microbial community (Liu et al., 2018). The root exudates of plants will be affected by biotic or abiotic factors, affecting the rhizosphere bacterial community (Medellin et al., 2017). Here, we investigated the effects of *Sphingomonas* sp. Cra20 on the rhizosphere indigenous bacterial community of *A. thaliana* under different water conditions by high-throughput sequencing. The results showed that the relative abundance of the bulk soil (CK) and plant rhizosphere (P) bacterial community varied greatly at the phylum level (Figure 9). We showed that the plant rhizosphere had significant selection and enrichment effects on the soil bacterial community, mainly because plant roots provide a source of exuded sugars, organic acids and other metabolites which can be used for bacterial nutrition and energy. Furthermore, root exudates play a role in recruiting bacteria that some better suited to exploit than others (Bulgarelli et al., 2013; Stringlis et al., 2018). The abundance of Actinobacteria was relatively higher in bulk soil than in the rhizosphere, and Proteobacteria was the most dominant phylum in the rhizosphere, and a similar result was found in *Arabidopsis* (Bulgarelli et al., 2012). The abundance of Bacteroidetes was higher in the rhizosphere than in bulk soil. Members of Proteobacteria and Bacteroidetes are characterized as copiotrophic soil bacteria that compete successfully only when organic resources are abundant (Fierer et al., 2007). However, the first 10 dominant bacteria accounted for more than 90% of the bacterial community in both bulk soil and the rhizosphere. In addition to the laboratory, in other habitats where the environments are different, the Proteobacteria and Actinobacteria are the most abundant taxa (Baquerizo et al., 2018).

Comparing the two aqueous conditions, the rhizosphere indigenous bacterial community changed between inoculation and non-inoculation after a long-time treatment (Figures 9, 10). The effect of drought on the rhizosphere bacterial community was relatively mild, but it was stronger on the endosphere than the rhizosphere bacteria, suggesting large indirect effects of drought through changes to the physiology or immune status in the host plant (Fitzpatrick et al., 2018). The inoculation treatment slightly changed the rhizosphere bacterial community under well-watered conditions, which might be due to the fact that *Sphingomonas* still plays a role, as we found that *Sphingomonas* had a higher abundance compared to non-inoculation after long-time treatment (Figure 9). Inoculated *Bacillus* spp. caused a high abundance of *Bacillus* in the rhizosphere bacterial community in the early stage, but the abundance of *Bacillus* decreased to the same level as the non-inoculated soil after several weeks. Moreover, the change in the rhizosphere indigenous bacterial community is not obvious (Qiao et al., 2017; Gadhave et al., 2018). However, inoculation with *B. amyloliquefaciens* FZB42 changed the endophytic bacterial community of sprouting broccoli (Gadhave et al., 2018). The rhizosphere bacterial community of *Arabidopsis* is changed if inoculated with *Pseudomonas simiae* WCS417 (Stringlis et al., 2018). This implies that the effectiveness of these strains relies on different mechanisms.

Based on the hierarchical clustering tree of the bacterial community composition, we found that WBW and WND had more similar rhizosphere bacterial communities (Figure 10), suggesting that inoculation with Cra20 might have a similar effect with WD treatment. Drought promotes the increase of LR density (Jupp and Newman, 1987). The rhizosphere bacterial community of WBD was different from others, which may be superimposed on the effects of both treatments (Figure 10). The surface area of LRs is relatively higher than that of the other root classes, which provides more root exudates and habitat in plant–microbe interactions. Furthermore, more LRs help plants to recruit more specialist microbial communities (Saleem et al., 2016, 2018). A reduction in plant biomass caused a loss in root exudates and had strong effects on the rhizosphere microbial community (Dey et al., 2012). Early or late growth stages of plants, which have different growth rates and substrate requirements, will influence the rhizosphere bacterial community (Yang et al., 2013). Furthermore, the length of the growth period has an important impact on plant biomass and rhizosphere bacterial community (Andreote et al., 2010; Yang et al., 2013). Therefore, the effect of Cra20 on *Arabidosis* growth and root system structure influenced the rhizosphere bacterial community.

There were more *Sphingomonas* in WBD, and we also found the abundance of *Burkholderia*, *Streptomyces* and *Mucilaginibacter* were higher in WBD (Figure 8C, 11). *Burkholderia* is one of the most commonly researched genera because of its excellent plant growth-promoting properties (Fernandez et al., 2012; Theocharis et al., 2012; Wang et al., 2015a,b). Drought also shifted the composition of the rhizosphere bacterial community, most notably by increasing the relative abundance of *Streptomyces*. A previous study found that *Streptomyces* isolated from wheat roots provide a potential benefit to host plants under drought stress, possibly through production of plant hormones and biochemical activities that help mitigate water stress (Yandigeri et al., 2012). *Mucilaginibacter* can produce indole-3-acetic acid and may have a role in promoting plant growth (Chimwamurombe et al., 2016). Plants will recruit beneficial bacteria that remain in the soil even when under stress and could enhance drought tolerance for other members of their species (Zolla et al., 2013). A recent study reports *P. simiae* WCS417 shapes the root microbiome assembly of *A. thaliana* by inducing roots to produce the antimicrobial coumarin scopoletin and excrete it into the rhizosphere (Stringlis et al., 2018). Inoculating exotic Cra20 might help plants to recruit beneficial bacteria. However, the mechanisms behind the microbially enhanced plant stress tolerance remain unclear because of the different functions of diverse PGPR strains; more research is needed on how plants recruit these beneficial bacteria.

## Conclusion

Our results showed that the PGPR *Sphingomonas* sp. Cra20 affected the morphological and physiological properties of *Arabidopsis*, promoted the formation of leaves, and influenced the development of the root system structure to increase water absorption capacity and alleviate the drought stress. Plants that exhibited a faster rate of development were bigger, but the reproductive time was not shortened. Furthermore, inoculation of Cra20 changed the rhizosphere indigenous bacterial community. Intriguingly, some of the bacterial taxonomic units with the most significant changes in relative abundance contain shown to promote plant growth in other studies. This growth-promoting effect will contribute to the development of agriculture in harsh environments. In addition, findings that inoculation accelerated growth without affecting the vegetative time may be new features of plant-bacteria interactions.

## Author Contributions

LA, YL, and HS conceived and designed the experiments. YL, FW, and JG performed the experiments. YL and TY collected the samples and data. YL, YH, FW, and MZ analyzed the data. HS, YL, and LA wrote the manuscript.

## Conflict of Interest Statement

The authors declare that the research was conducted in the absence of any commercial or financial relationships that could be construed as a potential conflict of interest.

## Footnotes

1 http://drive5.com/uparse/

2 http://rdp.cme.msu.edu/

3 https://www.ncbi.nlm.nih.gov/sra/

## ABBREVIATIONS

CB: inoculation-control
CFU: colony-forming units
CN: non-inoculation-control
LR: lateral root
MDA: malondialdehyde
MS: Murashige and Skoog
OTUs: operational taxonomic units
PGPR: plant growth-promoting rhizobacteria
POD: peroxidase
R2A: reasoner’s 2A
RH: root hair
SOD: superoxide dismutase
WBD: *A. thaliana* inoculation water-deficit
WBW: *A. thaliana* inoculation well-watered
WD: water-deficit
WND: *A. thaliana* non-inoculation water-deficit
WNW: *A. thaliana* non-inoculation well-watered
WW: well-watered
