# Supplementary figures and images for: Sphingomonas sp. Cra20 Increases Plant Growth Rate and Alters Rhizosphere Microbial Community Structure of Arabidopsis thaliana Under Drought Stress
Source: Front Microbiol. 2019 Jun 5;10:1221. doi: 10.3389/fmicb.2019.01221 (PMC6560172; doi:10.3389/fmicb.2019.01221)

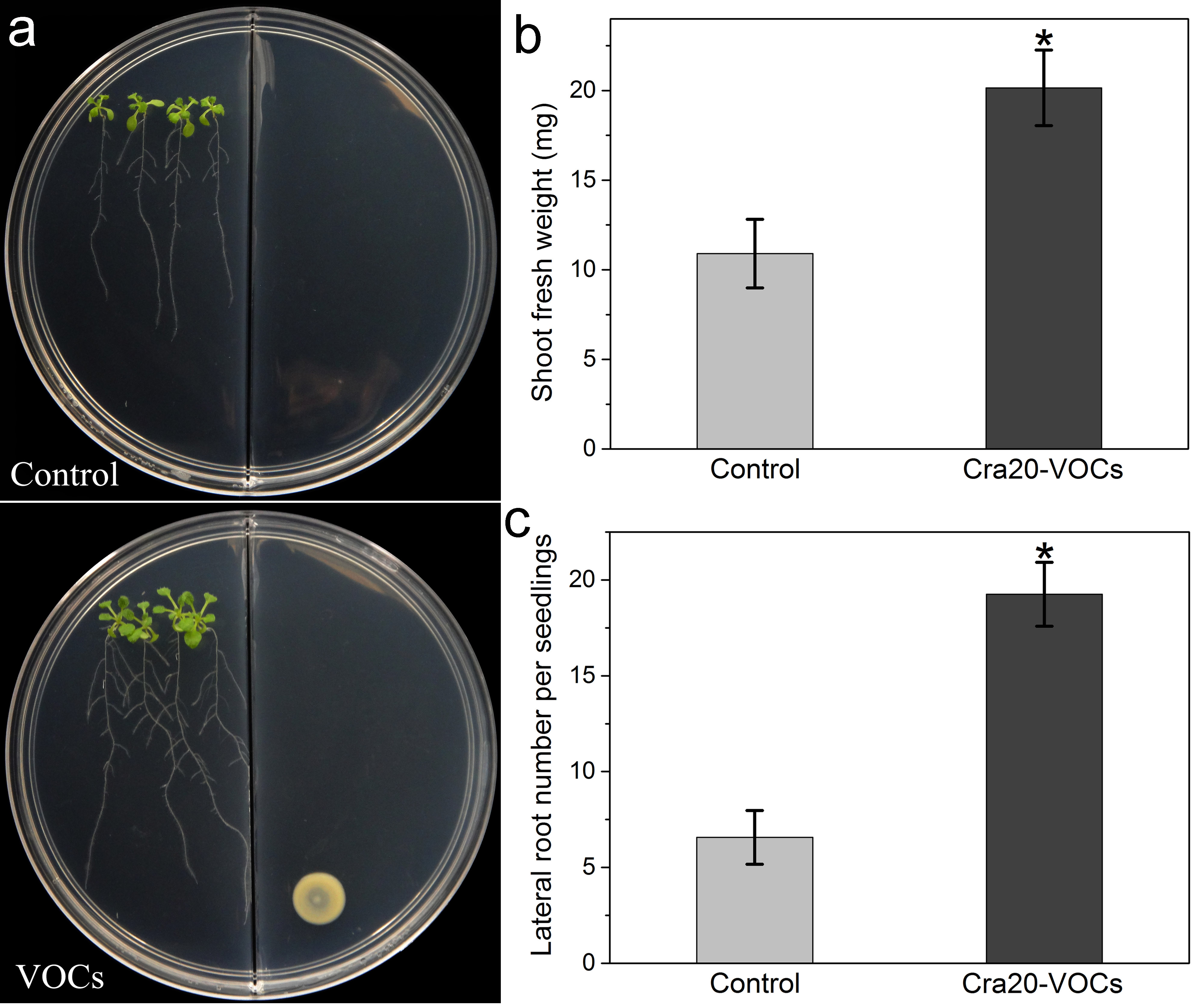

Supplement: Supplementary file 1 [file Image_1.JPEG]

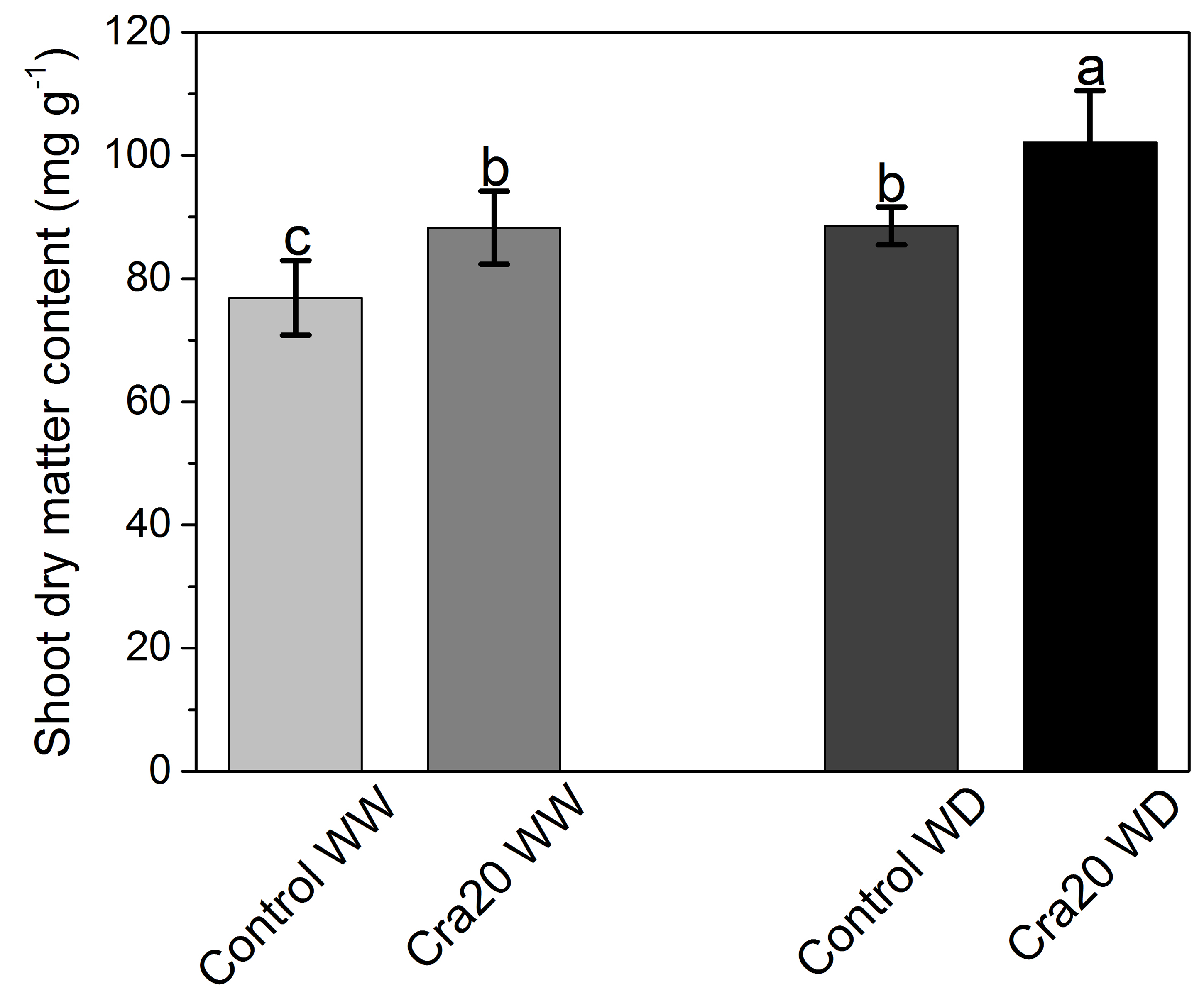

Supplement: Supplementary file 2 [file Image_2.JPEG]

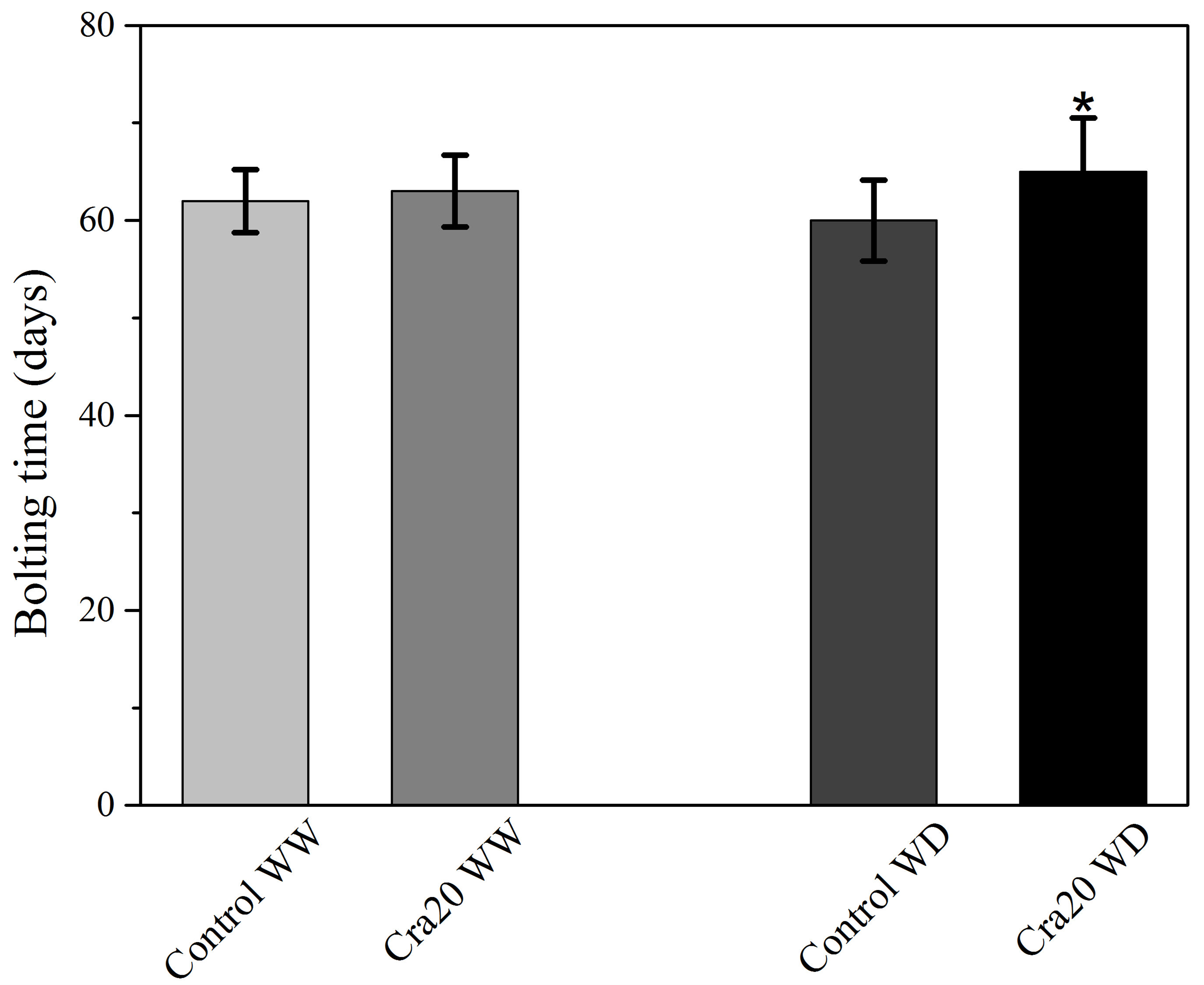

Supplement: Supplementary file 3 [file Image_3.JPEG]

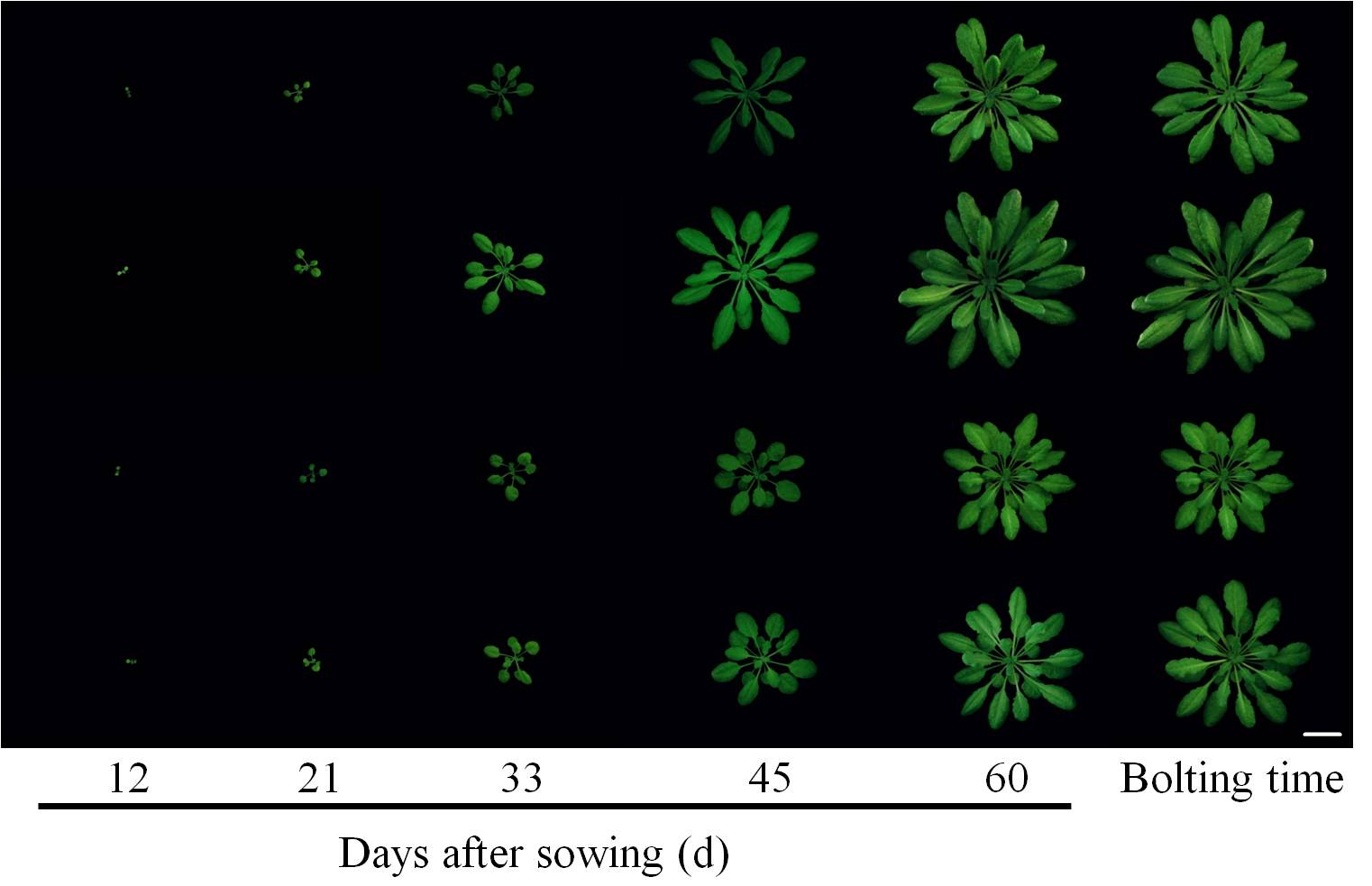

Supplement: Supplementary file 4 [file Image_4.JPEG]

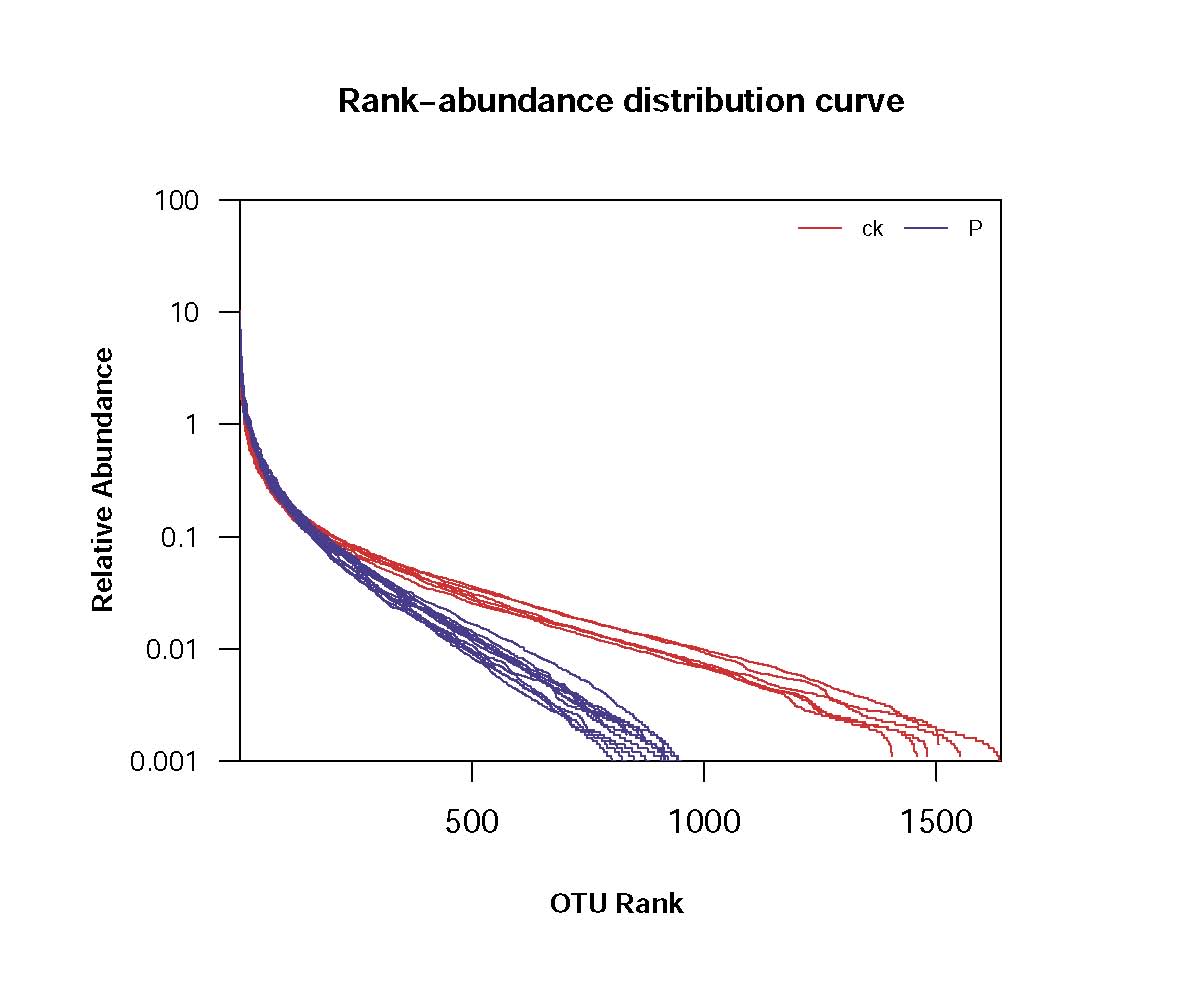

Supplement: Supplementary file 5 [file Image_5.JPEG]
