# Supplementary material for: Sphingomonas sp. Cra20 Increases Plant Growth Rate and Alters Rhizosphere Microbial Community Structure of Arabidopsis thaliana Under Drought Stress
Source: Front Microbiol. 2019 Jun 5;10:1221. doi: 10.3389/fmicb.2019.01221 (PMC6560172; doi:10.3389/fmicb.2019.01221)
Supplement: Supplementary file 6 [file Data_Sheet_1.docx]

Supplementary Material

***Sphingomonas* sp. Cra20 Increases Plant Growth Rate and Alters Rhizosphere Microbial Community Structure of *Arabidopsis Thaliana* Under Drought Stress**

Yang Luo^1^, Fang Wang1, Yaolong Huang^1^, Meng Zhou^1^, Jiangli Gao^1^, Taozhe Yan^1^, Hongmei Sheng^1^*, Lizhe An^1,2^*

^1^*Ministry of Education Key Laboratory of Cell Activities and Stress Adaptations,*

*School of Life Sciences, Lanzhou University, Lanzhou 730000, China*

^2^ *The College of Forestry，Beijing Forestry University, Beijing 100083, China*

***Corresponding author: Hongmei Sheng**

**Address: Tianshui Road 222, Lanzhou 730000, China**

**Tel: +86 181 5363 3997**

**E-mail:** [**shenghongm@lzu.edu.cn**](mailto:shenghongm@lzu.edu.cn)

***Corresponding author: Lizhe An**

**Address: Tianshui Road 222, Lanzhou 730000, China**

**Tel: +86 189 1988 9999**

**E-mail:** [**lizhean@lzu.edu.cn**](mailto:lizhean@lzu.edu.cn)

## Supplementary Figures and Table

**Supplementary Figure 1.** Plant growth promotion of *A. thaliana* seedlings by volatiles organic compounds (VOCs) from *Sphingomonas* sp. Cra20 or water (control). Plastic petri dishes that contained a center partition were used to test any mediated through soluble or volatile compounds produced by Cra20. **a)** Representative images of *A. thaliana* grown on a two partitioned plate system with or without Cra20. **b)** Shoot fresh weight measured after 7 d of co-culture with or without Cra20. **c)** Lateral root number per seedling. Data represent mean fresh weights ± SD of twelve groups of seedlings each consisting of two excised shoots. Lateral root number ± SD of at least 15 seedlings from five different plate. Asterisks indicate statistically significant difference compared with Control-treated roots (Student’s t test; *P* < 0.001).

**Supplementary Figure 2.** Effects of *Sphingomonas* sp. Cra20 and water-deficit on Leaf dry matter content of inoculated and non-inoculated *A. thaliana* under well-water (WW) and water-deficit (WD) conditions. Data are means ± SE of 9 plants. Different letters indicate significant differences following the ANOVA Duncan test at *P* < 0.05.

**Supplementary Figure 3.** Effects of *Sphingomonas* sp. Cra20 and water-deficit (WD) on bolting time of *A. thaliana*. Data are means ± SD of 30 plants (Student’s t test; *P* < 0.05).

**Supplementary Figure 4.** Representative images of *A. thaliana* in pot under different water conditions. Bar was 2cm.

**Supplementary Figure 5.** The Rank-Abundance distribution curve about the different treatments. The red line represent bulk soil (ck); the blue line represent plants rhizosphere (P).

**Supplementary Table 1.** Replicate numbers per trait and conditions of inoculated with Cra20 (B) and non-inoculated (N) plants under well-watered (WW) and water-deficit (WD).

|  | **Col-0 (Bolting)** | | | |
| --- | --- | --- | --- | --- |
|  | **WW** | | **WD** | |
| Trait | **B** | **N** | **B** | **N** |
| Shoot fresh weight | 9 | 9 | 9 | 9 |
| Shoot dry weight | 9 | 9 | 9 | 9 |
| Root dry weight | 9 | 9 | 9 | 9 |
| Phyllochron | 30 | 30 | 30 | 30 |
| Leaf number | 30 | 30 | 30 | 30 |
| Rosette leaf diameter | 30 | 30 | 30 | 30 |
| Total leaf area | 30 | 30 | 30 | 30 |
| LDMC | 9 | 9 | 9 | 9 |
| Proline | 6 | 6 | 6 | 8 |
| MDA | 6 | 6 | 6 | 8 |
| chlorophyll | 6 | 6 | 6 | 8 |
| POD activity | 6 | 8 | 8 | 8 |
| SOD activity | 6 | 8 | 8 | 8 |
| Rhizospheric soil | 9 (only soil) | 9 (only soil) | 9 (only soil) | 9 (only soil) |
| Control soil | 9 (another pots without plants) | | 9 (another pots without plants) | |

**Supplementary Table 2.** PERMANOVA analysis on rhizosphere bacterial community composition affect by *Sphingomonas* sp. Cra20 and water-treatment. **a)** All OTUs; **b)** All OTUs without *Sphingomonas*. Bact means *Sphingomonas* sp. Cra20 treatment; Treat means water treatment.

| **a.** All OTUs | | | | | | |
| --- | --- | --- | --- | --- | --- | --- |
|  | Df | Sums Of Sqs | Mean Sqs | F. Model | R^2^ | Pr (>F) |
| Bact | 1 | 0.2960 | 0.2960 | 3.6457 | 0.0663 | 0.0243* |
| Treat | 2 | 3.0352 | 1.5176 | 18.6919 | 0.6793 | 0.0001*** |
| Residuals | 14 | 1.1367 | 0.0812 |  | 0.2544 |  |
| Total | 17 | 4.4679 |  |  | 1.0000 |  |
| Signif. Codes: 0 '***' 0.001 '**' 0.01 '*' 0.05 '.' 0.1 ' ' 1 | | | | | | |
| **b.** All OTUs without *Sphingomonas* | | | | | | |
|  | Df | Sums Of Sqs | Mean Sqs | F. Model | R^2^ | Pr (>F) |
| Bact | 1 | 0.2903 | 0.2903 | 3.5549 | 0.0643 | 0.0250* |
| Treat | 2 | 3.0799 | 1.5400 | 18.8558 | 0.6824 | 0.0001*** |
| Residuals | 14 | 1.1434 | 0.0817 |  | 0.2533 |  |
| Total | 17 | 4.5137 |  |  | 1.0000 |  |
| Signif. Codes: 0 '***' 0.001 '**' 0.01 '*' 0.05 '.' 0.1 ' ' 1 | | | | | | |
